# Human Papillomavirus Infection: Knowledge, Risk Perceptions and Behaviors among SMW and AFAB

**DOI:** 10.3390/diagnostics12040843

**Published:** 2022-03-29

**Authors:** Magdalena Piróg, Bartosz Grabski, Robert Jach, Andrzej Zmaczyński, Magdalena Dutsch-Wicherek, Andrzej Wróbel, Klaudia Stangel-Wójcikiewicz

**Affiliations:** 1 Department of Gynecological Endocrinology and Gynecology, Jagiellonian University Medical College, 31-501 Krakow, Poland; robert.jach@uj.edu.pl (R.J.); dr.zmaczynski@gmail.com (A.Z.); klaudia.stangel-wojcikiewicz@uj.edu.pl (K.S.-W.); 2 Sexological Lab, Department of Psychiatry, Jagiellonian University Medical College, 31-066 Krakow, Poland; bartosz.grabski@uj.edu.pl; 3 Department of Psychiatry, Centre of Postgraduate Medical Education, 01-809 Warsaw, Poland; dutsch.wicherek@gmail.com; 4 Second Department of Gynecology, Medical University of Lublin, 20-090 Lublin, Poland; wrobelandrzej@yahoo.com

**Keywords:** HPV, cervical cancer, sexual minority women, people assigned female at birth

## Abstract

Human papillomavirus (HPV) infection is the most common sexually transmitted infection (STI) in the United States, and persistent HPV infection has been established as playing a major role in the development of cervical cancer. Providing HPV vaccination and regular screening tests have reduced the risk of developing cervical cancer or helped to detect the cancer at an early stage. Despite the above measures, cervical cancer still remains a major public health problem worldwide. Infection with HPV, and consequently cervical cancer, affects all people with an intact cervix, so not only heterosexual women, but also women from sexual minorities (SMW) together with people assigned female at birth (AFAB). These populations may be even more likely to develop cervical cancer, mainly because they are less likely to be aware of HPV transmission and prevention of cervical cancer. In our review, we summarized the current state of HPV knowledge, collected data assessing the orientation of this issue among SMW and AFAB, and indicated the causes of possible negligence in the prevention of cervical cancer.

## 1. Introduction

Human papillomavirus (HPV) infection is the most common sexually transmitted infection (STI) in the United States [1]. It is newly diagnosed among 7 million American women each year, especially between 16 and 25 years old [2]. HPV can be transmitted from sexual partners of any sex or gender as well as through skin-to-skin sexual contact and sharing fomites, i.e., sex toys during vaginal, anal and oral sex. Studies indicate specific genetic and behavioral factors increase the risk of HPV infection such as early age of sexual initiation, numerous sexual partners and an immunocompromised state among people after transplant therapy and with HIV infection. Persistent HPV infection is playing a major role in the development of lower genital tract precancerous and cancerous diseases. Each year, around 23,300 American women are diagnosed with HPV-related malignancy such as cervical cancer [1]. Providing HPV vaccination and regular screening tests has reduced the risk of developing cervical cancer or helped to detect the cancer at an early stage. Despite the above measures, cervical cancer still remains a major public health problem worldwide. 

HPV infection and, consequently, cervical cancer is indicated among all people with intact cervixes regardless sexual or gender identity The disease affects not only heterosexual women, but also people such as sexual minority women (SMW, e.g., lesbians, bisexual, queer women and women with same-sex sexual attractions or partners, regardless of their sexual identity) and people assigned female at birth (AFAB, e.g., nonbinary and trans-men) [3]. Evidence of the frequency of HPV infection in the AFAB population together with the incidence and prevalence of cervical cancer among SMW and AFAB is limited, because sexual and gender identity data are not collected in most cancer surveillance programs [4,5]. Analysis of available research has shown that HPV infection was confirmed among 36–53% of women with same-sex partners and 41–43.9% women with no same-sex partner [6].

Despite the similar prevalence of HPV infection among both sexual minority and heterosexual women, recent studies imply that SMW perceive themselves as less likely to acquire the HPV infection compared to all women. Moreover, these women may also receive limited cervical cancer prevention and screening. In this review, we try to gather and summarize the HPV-related knowledge and risk perceptions along with attitudes and behaviors among SMW and AFAB. Organized cervical cancer prevention and screening programs should consider the needs of these populations.

## 2. SMW and AFAB

### 2.1. HPV Knowledge

The United States includes at least four million SMW and around 150,000 youth (aged 13–17 years) along with 1.4 million adults (aged 18 years and older) identifying themselves as transgender [7]. Another study has shown that self-identified transgender and gender nonconforming individuals represented 0.1–2% of the global population [8]. The analysis of the data received from adolescent and adult residents of the Netherlands using an Internet-based survey showed that 0.2% respondents were AFAB [9]. In a similar study conducted among residents of the Flanders region in Belgium, AFAB constituted 0.6% [10]. A recent population-based study showed that among 50,157 Swedish adults, 2.3% of participants expressed feeling like someone of a different sex, and 2.8% wanted to live or be treated as a person of another sex [11]. Interestingly, interviews conducted among Taiwanese university students revealed that 7% of them are AFAB [12]. The literature describing the proportion of AFAB youth (younger than 19 years of age) in the general population is limited. A total of 0.65% (*n* = 96) of New Zealand school students described themselves as AFAB, and 1.5% (*n* = 121) reported that they were not sure [13]. Two surveys conducted among American youth showed that 0.56% (*n* = 17) from Boston and 1.8% (*n* = 1465) from Minnesota described themselves as AFAB [14,15]. Another analysis of data collected from public schools in San Francisco revealed that 1.3% of respondents considered themselves transgender, but the AMAB (assigned male at birth)/AFAB status was not provided [16]. Understanding the size of SMW and AFAB populations is a critical step in drawing attention to the needs of these societies along with establishing knowledge about cervical cancer prevention and screening.

#### 2.1.1. HPV Infection

There are over 150 genotypes of HPV. The 40 of them that infect the anogenital tract are divided into high-risk and low-risk groups (hrHPV and lrHPV, respectively), based on their oncogenic potential [17]. HPV16 and HPV18 are high-risk genotypes and are responsible for nearly 70% of high-grade cervical cancers in the US [18]. HPV infection is usually asymptomatic. Nearly 90% of all HPV viruses are either eliminated by immune systems or become inactive up to two years after infection [19]. Current data confirms that in most women, cervical cancer development was observed at least 3 to 5 years after hrHPV infection [20]. Persistent HPV infection is responsible for more than 5% of all cancers worldwide and more than 50% of all malignancies related to infection [21,22,23]. Therefore, early detection of both HPV infection and HPV-induced lesions are key to preventing the development of cervical cancer.

The prevalence of HPV in sexual and gender minority people with intact cervixes varies between 15 and 51%, and the prevalence is similar among heterosexual women [4]. Current studies have confirmed HPV infection among 58% of bisexual women, 36% of lesbians and 41% of heterosexual women [3]. Moreover, the national health and nutrition examination survey (NHANES) data confirmed the prevalence of HPV among women with same-sex partners as 51–53%, in contrast to 43.9% in women with no same-sex partners [4,5]. Moreover, the HPV-positive rate among lesbians in Western countries is estimated between 13–21%. Another study confirmed 25% of HPV-positive results among 300 investigated Chinese lesbians, and the hrHPV rate was 21.33%. Data describing the prevalence of HPV among AFAB are limited. We found only one study describing HPV prevalence among transgender men, showing 16% of hrHPV positive results in self-collected vaginal swabs [24]. 

#### 2.1.2. Cervical Cancer

The number of deaths caused by cervical cancer has decreased in the US from 2.8 per 1000 women in 2000 to 2.3 per 1000 in 2015 [25,26]. Around 99.7% cases of cervical cancer are associated with HPV infection. Cervical cancer is preventable through HPV vaccination, and regular cervical screening can detect the cancer at an early stage. However, most cases of cervical cancer deaths occur among inadequately screened women [25]. The incidence and prevalence of cervical cancer among SMW and AFAB populations are both limited since gender and sexual identity data are not collected in most cancer surveillance programs [6]. Only one study showed that that sexual minority women had increased rates of cervical cancer diagnosis compared to heterosexual women [4].

#### 2.1.3. HPV Vaccine

The Centers for Disease Control and Prevention (CDC) recommends routine HPV vaccination at ages 11–12 for all sexes, however it can be started as early as age 9 [27]. In public health programs, HPV vaccine is recommended for people under age 25 at the initiation of vaccinations. However, some scientific societies (i.e., Centers for Disease Control and Prevention, American Cancer Society) allow HPV vaccination in older patients if the benefits outweigh the risks related to the vaccine. HPV vaccines can be administered in two different regiments—two doses and three-doses, depending on the age at initiation of vaccination and immune status [28,29] (Table 1). Since 2016, only 9-valent HPV vaccine (against HPV types 6, 11, 16, 18, 31, 33, 45, 52 and 58) has been available for use in the US [29]. Moreover, the HPV vaccine may be given at the same time as other vaccines. Recent studies have confirmed HPV vaccine uptake of 10–18.2% among SMW women [30,31,32]. These low rates of vaccination were similar across all subsamples: lesbian (20.8%, *n* = 40) and bisexual women (30.8%, *n* = 95) [33]. It might be speculated that perceptions of low HPV risk are responsible for lower HPV vaccine rates among lesbians when compared with heterosexual women, as well as among women with only female sexual partners in contrast to women with male sexual partners [1]. There are currently no data available on the number of AFAB who have received the HPV vaccine.

#### 2.1.4. Cervical Screening and HPV Screening

The Papanicolaou (Pap) test is an important cancer screening procedure for people with intact cervixes. It allows health care providers to detect precancerous and cancerous lesions caused by HPV infection. Current data indicate the lack of clinically important differences between conventional and liquid-based cytology (LBC) [34]. According to the American College of Obstetricians and Gynecologists (ACOG) recommendations, cis-gender women (women whose gender identity aligns with the sex assigned to them at birth). along with AFAB individuals with intact cervixes, should follow the same screening guidelines as heterosexual women [7,35]. Cervical cancer screening should be performed per age-related guidelines for all people with intact uteruses [26,36] (Table 2). However, certain risk factors such as a compromised immune system or previous treatment of a high-grade precancerous stage may increase cervical cancer risk. Therefore, people with these factors should receive individualized follow-up. Additionally, it is worth paying attention to the particular circumstances relating to the AFAB population, such as future plans for cervix removal. Permanent sterilization, defined as the definitive removal of the reproductive organs, used to be listed as an obligatory step to change legal gender, and it is still mandatory in Japan and in some American states. Growing acceptance of transgender people may lead to less restrictive laws and two important conditions. First, as legal requirements for hysterectomies are not necessary, we can expect a growing population of transgender men with intact cervixes will need cervical screening. Second, while some transgender men who will retain their cervix remain registered legally as women, others will be registered as men and may not be able to take part in the national cervical cancer screening program. The US transgender survey reports that 71% of transgender men (*n* = 7950) have received androgen therapy and 14% have had hysterectomies [37]. Another problem with the AFAB population, especially transgender men, is cervical and vaginal atrophy secondary to testosterone, which may hamper the cervical cancer screening. Trans-male individuals have been confirmed to have a 10-times higher rate of unsatisfactory cytological results, in contrary to cis-genders [38]. However, the recent study on 84 transgender men showed that they were not at a higher or lower risk of abnormal Pap test results compared with women [39]. Nevertheless, larger studies are needed to support above findings.

A HPV test is performed in the same way as a Pap test to determine the risk of cervical infection by hrHPV genotypes. The American Cancer Society recommends it in individuals with cervixes at ages 25 to 65. The ACOG states that self-collected HPV specimens may be appropriate for those patients who do not have access to screening or for whom speculum insertion is physically difficult or may be emotionally traumatic. Nevertheless, there is no self-collected HPV test approved by the U.S. Food and Drug Administration (FDA). AHPV test should be performed individually every 3 years or together with a Pap test (called a co-test) every 5 years.

### 2.2. Risk Perceptions

Risk perceptions play a key role in creating health behaviors. Based on the available literature, it is slightly different in the discussed populations in terms of both HPV transmission and vaccine.

#### HPV Transmission

Several studies have confirmed that lesbians and women with only AFAB partners tend to perceive themselves as less likely to acquire HPV than bisexual women and (AMAB) [4,40,41,42]. Moreover, studies have shown that SMW who have sex with bisexual women and AFAB individuals who have AMAB partners have a notable HPV risk factor [43]. Interestingly, participants’ HPV risk perceptions do not vary by race or ethnicity [32]. Marazzo et al. reported that young adult lesbian and bisexual women believed that female-to-female HPV transmission was decreased when compared to heterosexual intercourse [44]. Other studies showed that only 59.6% of Italian [2] and 44.8% of Australian [45] participants, including lesbian, gay men and bisexuals, have heard about HPV infection. In contrast, 79% of gay and 93% of bisexual Americans reported hearing about HPV [46,47]. Data on the perception of HPV transmission among AFAB is scarce. One of the studies showed that the majority of sexually active AFAB individuals was aware of being at some risk of acquiring HPV, regardless of gender and sexual identity. However, based on survey data, a hierarchy of perceived HPV risk emerged based on sexual behavior and sexual identity. Precisely, most of the participants believed that the highest risk of HPV was among those who have penile-vaginal sex. The risk of acquiring HPV from AFAB sexual partners was perceived incorrectly as low or negligible [39]. Several studies confirmed that most participants erroneously attributed the risk of HPV transmission in the context of sex among AFAB individuals to the exchange of genital fluids rather than to skin-to-skin sexual contact [39,47,48]. Some researchers have suggested that the misconceptions about the risk of transmitting HPV may be due to comparing the acquisition of this infection to sexually transmitted infections via body fluids (e.g., HIV) [48,49].

Consequently, low HPV risk perceptions are also responsible for less safe -sex practices that include not limiting the number of sexual partners and reduced use of barrier methods among SMW and AFAB. Nevertheless, the widespread occurrence of HPV carries the risk of infection even after one intercourse. Moreover, the use of condoms reduces but does not eliminate, the risk of HPV infection because the virus can be transmitted from skin not covered by the condom that must be used from the beginning to the end of intercourse [2]. These misperceptions may undermine individuals’ engagement in HPV prevention strategies, including HPV vaccination and cervical screening.

### 2.3. Attitudes and Behavior

#### 2.3.1. HPV Vaccine

It has been suggested that perceived HPV risk was positively associated with HPV vaccination among American young adults [1]. With regard to the level of HPV vaccine knowledge from recent studies, only 42.1% of Italian participants reported that they were aware of available protection against HPV. Higher awareness of HPV vaccine has been observed among Australian lesbians and bisexual women (50% and 54.6%, respectively). Currently, there are no data reporting knowledge about both HPV infection and vaccine among AFAB.

Current data on the initiation and completion of HPV vaccination in different sexual orientation groups are inconsistent. Several studies suggest that lesbians are less likely to vaccinate against HPV than heterosexual women. However, there were no difference in initiation of HPV vaccination between bisexual and heterosexual women [1,31,39]. In contrast, other researchers have shown that both lesbians and heterosexual women are equally likely to initiate and complete HPV vaccination, whereas bisexual women are more likely to receive the complete course of HPV vaccination than other women are [50,51]. Another study suggested that women experience similar protection against HPV infections, regardless of sexual orientation [52]. This finding might be explained by the fact that the HPV vaccine is normally administered prior to the development of sexual orientation. Nevertheless, there are no HPV vaccine recommendations to sexual and gender minority people or parents of SMW and AFAB adolescents defining a vaccination regimen determined by the age of the patient.

#### 2.3.2. HPV/Cervical Screening

Even in the relatively extensive literature on sexual identity and cytology tests, studies assessing the frequency of cervical screening in people from sexual minorities are limited. According to current data, only 10–12% SMW continue regular cervical screenings [53]. Another study has shown that during the three years before the study, 48–81% of American lesbians reported having a Pap test, around 57% in the United Kingdom and 78% in Australia [54]. The study also confirms that cervical screening among lesbians is 5–18% lower than in heterosexual women. Moreover, there is no data determining the age at which these individuals have their first Pap tests or HPV test. Taking the Pap test too late together with not performing screening for hrHPV may put both SMW and AFAB at higher risk of HPV complications such as late diagnosis and poorer cancer outcomes [55]. Nevertheless, most of the guidelines still do not specify cervical cancer screening recommendations for sexual and gender minorities, which is crucial to reduce the cervical cancer risk. Moreover, to the best of our knowledge, the progress of dysplasia and cancer genesis among SMW and AFAB has not yet been described in the literature.

## 3. Cervical Cancer Screening Barriers for Sexual Minority People (SMW and AFAB)

**Figure 1:**
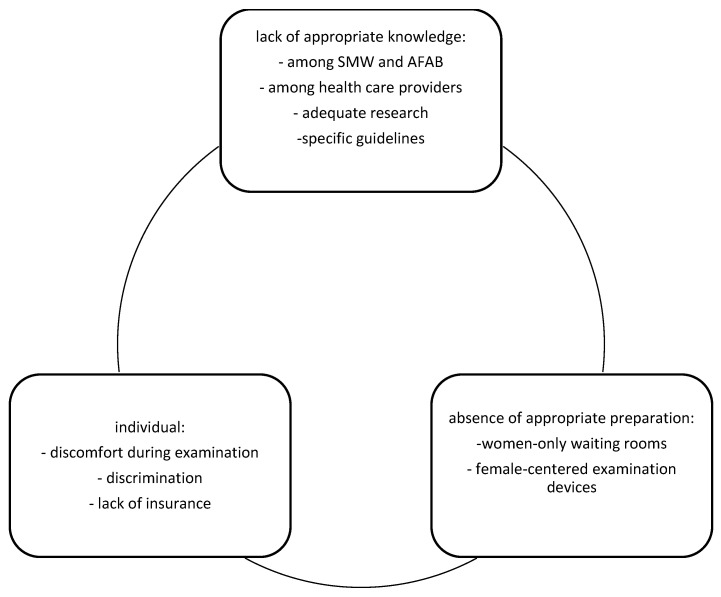
Cervical cancer screening barriers for SMW and AFAB.

We have described the barriers that SMW and AFAB face in accessing cervical screening (Figure 1). Reduced cervical cancer screening may have a multifactorial background.

First, lack of adequate knowledge indicates several levels—interested individuals, health care providers, appropriate research and guidelines. Several studies showed that sexual minority women believed that they do not need screening if they did not have sex with men [56,57]. On the other hand, further data reported that most participants believed that HPV and cervical cancer risk did not differ by gender or sexual identity, but they could not answer how often cervical cancer screening should be performed [58]. In contrast, some believed that testosterone use increased their risk of cervical cancer [59]. Moreover, beliefs in low HPV risk led to reduced use of barrier methods such as condoms [58,60]. Taken together, the data confirm a lack of consensus among participants about how their gender minority status influenced both cervical cancer risk and therefore screening [59]. Furthermore, access to health care providers may also shape patterns of cervical cancer screening behavior [61,62]. Receiving adequate information about HPV transmission, together with cervical cancer prevention and screening, may lead to the opposite situation. Reduced need for contraception limits necessity of medical visits [63,64]. Moreover, restricted knowledge about HPV transmission and risky sexual behavior may arise from misinformation and lack of proper training among health care providers [54,60]. According to US data, only 20% of 141 gynecology trainees received postgraduate training on the care of gender minority patients [61]. When asked particularly about care among gender minorities, 89% admitted that cervical cancer screening should be performed, but only 29% felt confident performing the screening [54,62]. Possible explanations may be either the lack of proper education in how to perform the examination or psychological barriers to including ‘female’ designated procedures if the patient has already changed their legal gender [54,63]. Lack of knowledge about the examination technique was confirmed by several studies [1,63], and in addition, some care providers reported that if the patient had lower sexual risk and was waiting for a total hysterectomy, screening could be postponed or avoided [40]. Moreover, lack of knowledge among health care providers arises from the limited relevant research concerning the course of HPV infection and cervical cancer development among the abovementioned populations. The lack of scientific outcomes and therefore of suitable guidelines lead to the neglect of the different conditions and needs of SMW and AFAB in both cancer screening and prevention programs.

Second, is the lack of preparation for the cervical cancer screening. Starting from women-only waiting rooms and female-concentrated tools for cervical cancer screening, such as specula, most of the health care centers are not adequately prepared to provide services for these people.

Additionally, one of the studies showed that many AFAB do not identify as women. Therefore, the speculum is the most off-putting aspect of a cervical examination, and consequently, application of the conventional Pap smear is restricted [65]. That is why one of the studies emphasized the need to provide alternative screening options for sexual minorities [24] that were also considered in other studies [51,65,66]. Interestingly, another research with 131 gender minority participants compared a conventional Pap test with hrHPV DNA hybridization assays from both self-and provider-collected vaginal swabs [24]. In contrast to Pap tests, self-collected vaginal specimen assays for hrHPV had a sensitivity of 71% and a specificity of 98%, with substantial concordance between techniques (κ = 0.75; *p* < 0.0001), whereas in the provider-collected vaginal swab, stated to be the gold standard, the DNA assay conducted on the self-collected vaginal specimens had 86% sensitivity and 98% specificity, with near perfect concordance (κ = 0.84; *p* < 0.0001). Moreover, self-collected HPV swabs were considered by participants less inconvenient than the conventional Pap test [67]. Another study confirmed that among cisgender women (nontransgender), self-swab HPV-DNA testing as a primary cervical cancer screening method and self-swab specimen collection for other STIs have sufficient level of acceptance [68]. Therefore, it might be speculated that use of cotesting, including Pap smear and HPV DNA testing, every 5 years as per heterosexual women, might be both the most effective and the most acceptable method of cervical cancer screening among sexual and gender minority people, including trans and nonbinary individuals experiencing gender dysphoria (GD).

Finally, individual barriers are tied to discomfort resulting from the examination, discrimination and lack of insurance. Sexual and gender minority people such as AFAB are more likely to experience discrimination in health care, which may explain sexual and gender orientation-related health disparities [50]. Therefore, the social and economic consequences of living with a stigmatized sexual and gender identity are thought to explain sexual and gender orientation-related health disparities. Moreover, in many countries, lack of insurance covering gender-affirming surgical procedures or participation in free cervical cancer screening program for female members may also lead to the lack of appropriate cervical screening.

## 4. Conclusions

In conclusion, general knowledge and risk perceptions in HPV transmission among SMW and even more with AFAB is insufficient. In our review, we summarized the current state of HPV knowledge, collected data assessing knowledge of this issue among SMW and AFAB, and indicated the causes of possible negligence in the prevention of cervical cancer. Taken together, our results should draw the attention of many populations, not only medical ones, to the problems related to HPV infection among the discussed populations. This knowledge might also lead to the implementation of targeted intervention programs for the mentioned populations. Moreover, they can also provide parents and caregivers with the knowledge and communication skills they need to deliver accurate information about HPV risk and prevention to their adolescent and young adult family members from sexual and gender minorities.

## Figures and Tables

**Table 1:** HPV vaccine regiments (for ninevalent vaccine) [28].

Recommended Number of Doses	Recommended Dosing Schedule	Population
1	0 and 6–12 months (minimum interval between the doses: 5 months)	At ages 9–14 years at initiation of HPV vaccine, but not immunocompromised persons
3	0, 1–3, 6 months (minimum interval between the 1st and the 2nd dose: 4 weeks; minimum interval between the 2nd and the 3rd dose: 12 weeks; minimum interval between the 1st and the 3rd dose: 5 months;)	At ages 15–26 years at initiation of HPV vaccine OR At ages 9–26 years at initiation of HPV vaccine in immunocompromised persons OR At ages 27–45 years at initiation of HPV vaccine

**Table 2:** Cervical screening recommendations [26,36].

	Women Aged 21–29 Years	Women Aged 30–65 Years	Women Younger That 21 Years, Women Older than 65 Years and Women Who Have Had Hysterectomy with Cervix Removal
Recommendation	Screen for cervical cancer every 3 years with cervical cytology alone	Cervical cytology alone every 3 years OR hrHPV testing alone every 5 years OR Cervical cytology and hrHPV testing (cotesting) every 5 years	Do not screen for cervical cancer
